# New high-resolution prototype versus standard spectralis optical coherence tomography in patients with central serous chorioretinopathy

**DOI:** 10.1186/s40942-024-00598-6

**Published:** 2024-10-16

**Authors:** Lorenzo Ferro Desideri, Luc Hennebert, Yousif Subhi, Martin Zinkernagel, Rodrigo Anguita

**Affiliations:** 1 Department of Ophthalmology, Inselspital, Bern University Hospital, University of Bern, Freiburgstrasse 15, Bern, CH-3010 Switzerland; 2 Department for BioMedical Research, University of Bern, Murtenstrasse 24, Bern, CH-3008 Switzerland; 3 Bern Photographic Reading Center, Inselspital, Bern University Hospital, University of Bern, Bern, Switzerland; 4 Faculty of Medicine, University of Bern, Murtenstrasse 11, Bern, CH-3008 Switzerland; 5 Department of Clinical Research, University of Southern Denmark, Odense, Denmark; 6 Department of Ophthalmology, Rigshospitalet, Glostrup, Denmark; 7 Department of Ophthalmology, Zealand University Hospital, Roskilde, Denmark; 8 Moorfields Eye Hospital , NHS Foundation Trust, EC1V 2PD London, United Kingdom

**Keywords:** Central serous chorioretinopathy, CSC, High-res OCT, Standard OCT, Biomarkers, Acute CSC, Chronic CSC

## Abstract

**Purpose:**

To assess the accuracy of High-Resolution OCT in detecting biomarkers associated with central serous chorioretinopathy (CSC) compared to standard OCT.

**Methods:**

We conducted a cross-sectional study involving CSC patients who underwent High-Resolution and standard OCT during the same visit. Using the SPECTRALIS High-Res OCT device (Heidelberg Engineering, Heidelberg, Germany), macular B-scans were obtained and compared with those acquired using a SPECTRALIS HRA + OCT device (Heidelberg Engineering, Heidelberg, Germany). Qualitative assessments were performed, and statistical analyses compared the performance of both OCT modalities.

**Results:**

Thirty-one patients diagnosed with CSC were included with a mean age of 56.3 years (± 10.2). Among them, 29% (*n* = 9) were classified as acute CSC (aCSC), while 71% (*n* = 22) had chronic CSC (cCSC). High-Resolution OCT outperformed standard OCT in detecting microstructural changes in the outer retinal layers, including a higher prevalence of disrupted interdigitation zone (IZ) (29% vs. 6%, *p* = 0.003) and retinal pigment epithelium (RPE) disruption (12% vs. 2%, *p* = 0.0024). Intergrader agreement was high (Cohen’s Kappa = 0.85).

**Conclusion:**

High-Resolution OCT demonstrates promise in identifying critical biomarkers associated with CSC, particularly disruptions in the IZ and RPE. Further validation in larger cohorts is required to confirm their clinical relevance in patients with CSC.

## Introduction

Central serous chorioretinopathy (CSC) is characterized by the serous neurosensory detachment due to the accumulation of subretinal fluid (SRF), increased choroidal hyperpermeability and thickened choroid [1]. CSC is traditionally categorized into two subtypes: acute (aCSC) and chronic (cCSC) [2]. Typically, aCSC resolves spontaneously within 3 to 6 months, whereas cCSC is distinguished by the persistence of subretinal fluid (SRF) for longer durations, often exceeding 4 to 6 months [1, 3]. While the classification of CSC benefits from multimodal imaging including fundus autofluorescence (FAF), fluorescein angiography (FA), and indocyanine green angiography (ICGA), the introduction of optical coherence tomography (OCT) in clinical practice has significantly enhanced the ability to detect retinal microstructural changes and qualitative variations in retinal layers occurring along the clinical course of the disease [4–6].

In addressing this need, the recent advancements in OCT technology have been crucial. The standard spectral-domain OCT, with its 880 nm center wavelength and 40 nm spectral bandwidth, provides an axial resolution of approximately 7 μm in the eye [7]. By contrast, the new High-Res-OCT SPECTRALIS operates at an 840 nm central wavelength with a broader 130 nm bandwidth, boosting the axial resolution from 7 to 3 μm without exceeding laser exposure limits [8].

To date, no study has investigated the accuracy of High-Res OCT in examining patients with CSC. Therefore, our study aims to address this gap by comparing High-Res-OCT with the standard spectral-domain OCT in patients with CSC and exploring its potential additive value in this patient population.

## Methods

### Study subjects and design

This is a prospective single-center cross-sectional clinical case series of patients with diagnosis of CSC at the Inselspital, Bern University Hospital in Switzerland. The study was conducted in accordance with the Swiss Human Research Act, the ICH guidelines of Good Clinical Practice, the Declaration of Helsinki and was authorized by the local ethics committee (ID-2023-00768). Written informed consent was obtained from each participant.

The diagnosis of CSC was based on a combination of clinical history, examination, and various imaging techniques, such as OCT, fundus autofluorescence (FAF), fluorescein angiography (FA), and indocyanine green angiography (ICGA) and OCT angiography (OCTA) in cases of choroidal neovascularization (CNV) suspect. Patients with CSC were categorized into two groups: aCSC if SRF resolved spontaneously within 4–6 months from symptom onset, and chronic CSC cCSC if SRF persisted beyond 6 months. This classification was supported by findings from multimodal imaging.

Exclusion criteria included other macular diseases, previous interventions like anti-VEGF injections due to secondary choroidal neovascularizations, vitreoretinal surgery history, uveitis, glaucoma, optic nerve disease, high myopia, and a family history of systemic diseases affecting the macula. All participants underwent during the same day a full ophthalmic examination, including best corrected visual acuity (BCVA) measurement, intraocular pressure measurement, slit-lamp biomicroscopy, and dilated posterior segment examination with indirect ophthalmoscopy.

All the patients underwent OCT imaging (both standard SD-OCT and High-Res OCT) during the same visit and same part of day (in the morning) to avoid diurnal variations of choroidal and retinal layers [9].

### Standard spectralis HRA-OCT

The Standard Spectralis HRA-OCT imaging system was adopted (Heidelberg Engineering Inc., Heidelberg, Germany). OCT volumes covering an area of 5.90 mm × 5.75 mm × 1.92 mm centered on the fovea with a 49 B-scan acquisition protocol and a resolution of 496. To mitigate the influence of patient fixation variability on image quality, 25 automatic-real time (ART) frames were utilized consistently. The scan area was set for a volume of 20°× 20° OCT scan analouglsy to the High-Resolution modality.

### High-resolution spectralis OCT

High-Resolution OCT (Heidelberg, SPECTRALIS^®^ High-Res OCT- DMR001) was performed with a scan protocol of 20° × 20° OCT volume scan in high-resolution mode centered on the fovea with 49 B-scans and 25 ART frames (1024 a-scans per b-scan). High-Resolution OCT operates on the principles of Spectral Domain OCT, similarly to the standard Spectralis HRA-OCT, with a notable refinement in axial resolution within tissue, achieving a 3 μm resolution as opposed to the 7 μm standard platform. Further details can be found our previous the study by Reche et *al.* [8].

Patients with poor image quality and/or artifacts were excluded from the analysis.

### OCT qualitative assessment

As reported in previous studies analyzing OCT biomarkers in CSC, the following OCT qualitative parameters were evaluated: disruption of external limiting membrane (ELM), disruption of ellipsoid zone (EZ), disruption of interdigitation zone (IZ), disruption of RPE and Bruch’s membrane complex, presence of foveal bulge, presence of flat irregular pigment epithelium detachment (PED) and dome-shaped PED, fibrin exudation, presence of high refractive outer nuclear layer (ONL) band, hyperreflective foci (HF) in the ONL and in the choroid, distinction of choroidal vessel walls and central macular edema (CME), defined as the presence of oval hyporreflective areas larger than 100 μm diameter between the internal limited membrane and photoreceptor layer in macular region [4, 10]. Two expert ophthalmology specialists (RA and LFD) independently graded the OCT images, and intergrader agreement was calculated.

The performance in detecting the presence of these OCT parameters was compared between the Standard Spectrails OCT and High-Res OCT.

### Statistical analysis

The statistical analyses were performed with Statistical Package for Social Sciences; version 15.0. A Shapiro-Wilk test was performed for all variables to detect departures from a normal distribution. Continuous variables were expressed as the mean ± standard deviation and categorical variables were expressed as a percentage (%). Given the normal distribution of the data, a Fisher test was used to evaluate the associations between categorical variables. Cohen’s kappa coefficient was calculated to measure intergrader agreement performance. Power analysis of sample size was performed. A value of *p* < 0.05 was considered statistically significant.

## Results

### Demographic and clinical features

Thirty-one patients were recruited, whose 29% (*n* = 9) were diagnosed with aCSC and 71% (*n* = 22) with cCSC. The mean age of the population at the moment of the study was 56.3 years (± 10.2) and 19.4% (*n* = 6) were of male sex and 80.6% (*n* = 25) of female sex. The mean best-corrected visual acuity (BCVA) was 0.14 LogMAR (± 0.07) at the time of the visit.

None of the participants had a history of smoking; however, 12.9% (*n* = 4) reported previous corticosteroid use. Regarding treatment history, 45.2% (*n* = 14) of participants, all within the chronic group, had undergone photodynamic therapy (PDT) on average 11.2 (± 5.3) months before the visit, while 54.8% (*n* = 17) where treatment naive. Demographic features are listed in the table (Table 1).

**Table 1:** Demographic features of the patients with central serous chorioretinopathy enrolled in the study

Demographic features	
Mean Age (±)	56.3 years (± 10.23)
N Acute (%)	29% (*n* = 9)
N Chronic (%)	71% (22)
N Right eye (%)	61.3% (*n* = 19)
N Left eye (%)	38.7% (*n* = 11)
N Males (%)	6 (19.4)
N Females (%)	25 (80.6)
N Smoking History (%)	0% (*n* = 0)
N Corticosteroid use (%)	12.9% (*n* = 4)
Mean LogMAR BCVA (±)	0.14 (± 0.07)
N No PDT before visit (%)	54.8% (*n* = 17)
N PDT before visit (%)	45.2% (*n* = 14)

BCVA = best-corrected visual acuity; PDT = Photodynamic therapy

### OCT analysis

Power analysis, with an α value of 0.05, demonstrated that the current sample size (*n* = 31; 9 acute CSC, 22 chronic CSC) is sufficiently powered to detect large effect sizes (Cohen’s d ≥ 1.2.)

**Fig. 1:**
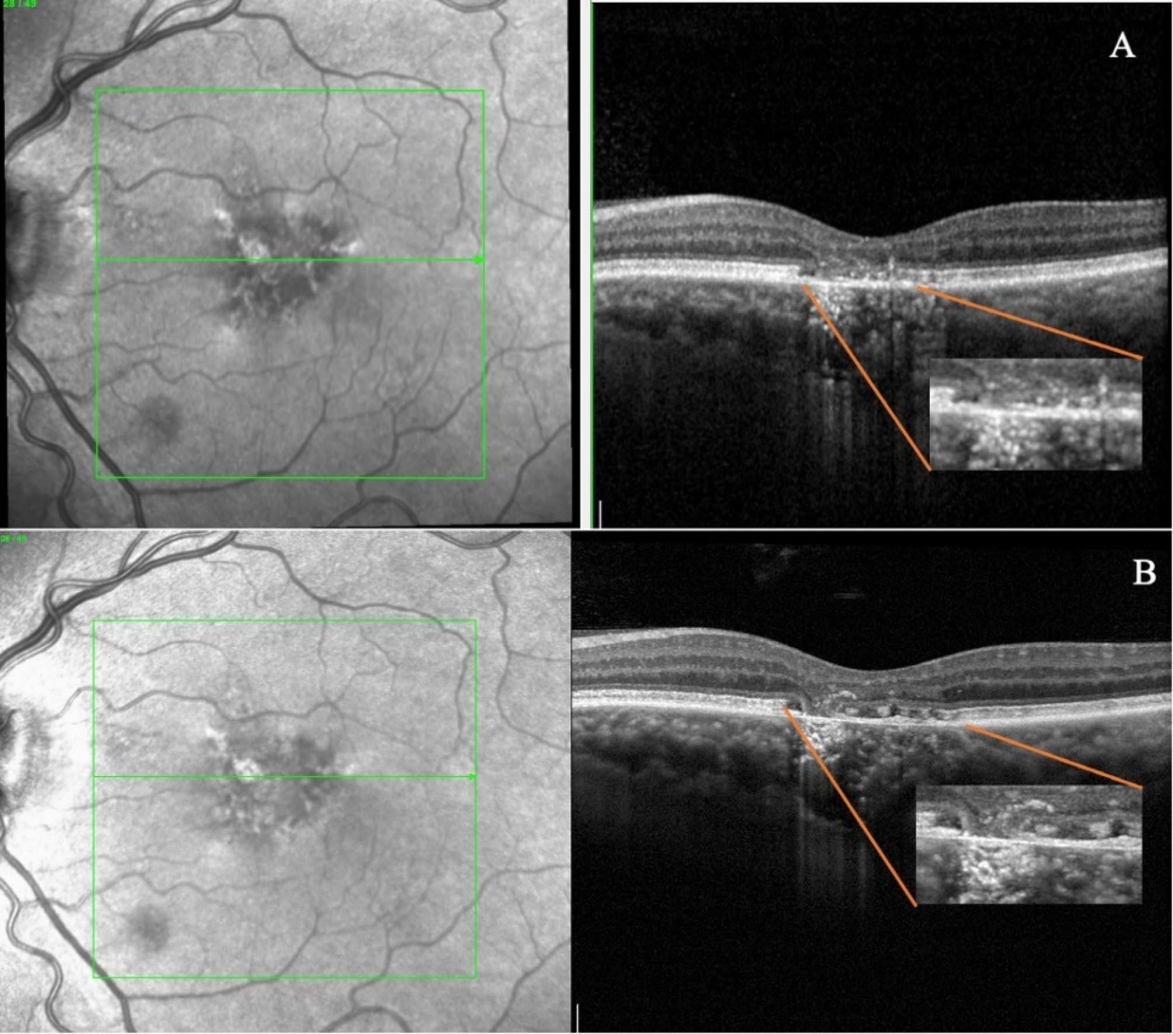
Comparison of interdigitation zone discernibility between the standard OCT **(A)** and High-Resolution OCT **(B)** in a patient with chronic central serous chorioretinopathy. With this latter modality it is possible to describe the focal interruption of the IZ layer **(B)**, which cannot be clearly distinguish with standard acquisition **(A)**

**Fig. 2:**
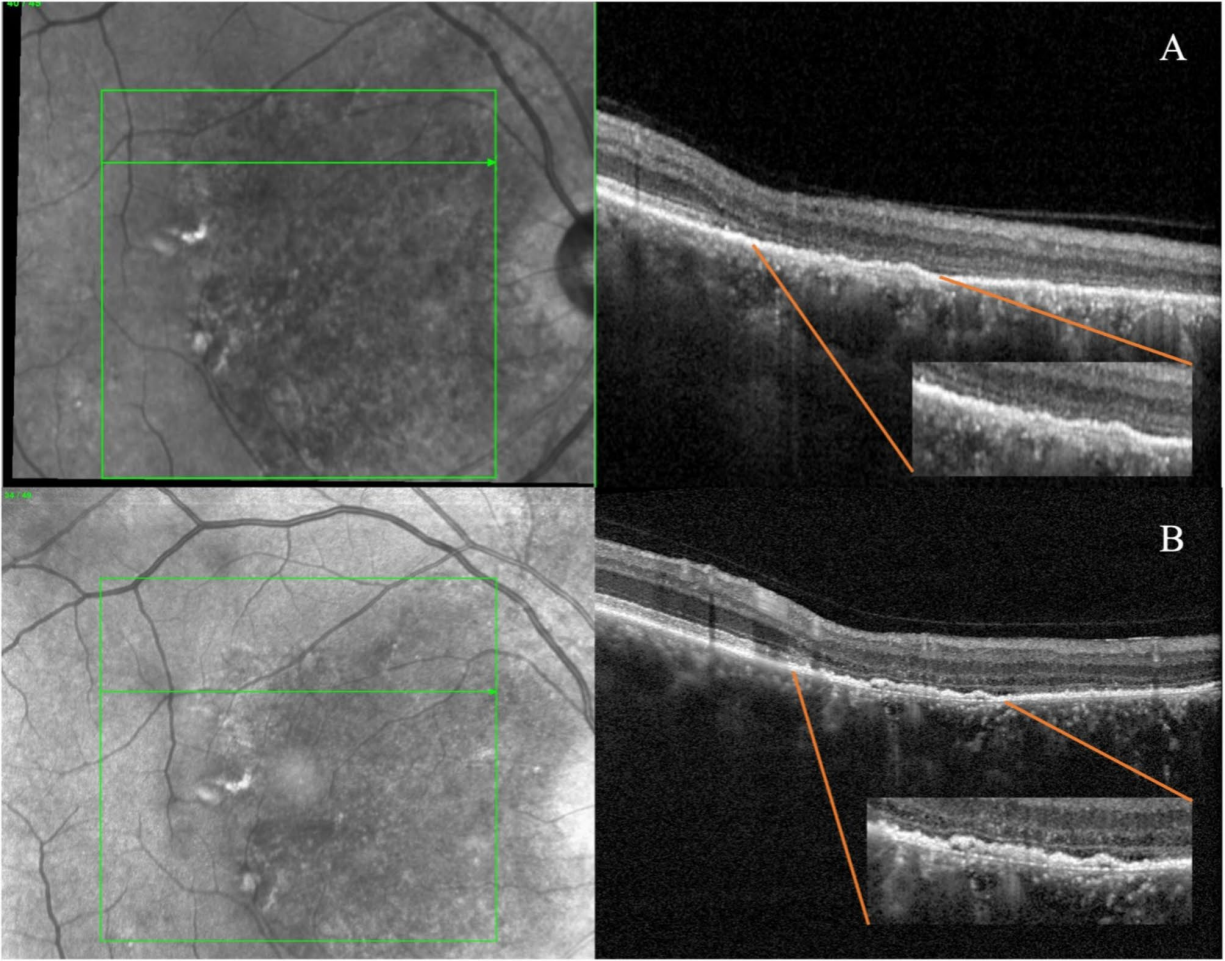
Comparison of retinal pigment epithelium focal disruption discernibility between the standard OCT **(A)** and High-Resolution OCT **(B)** in a patient with chronic central serous chorioretinopathy. Focal interruption of RPE disruption with altered reflectivity is visible in the High-Resolution acquisition **(B)**, whereas these changes are not detectable by standard OCT acquisition with the RPE appearing as a uniformly reflective band due to the lower definition of the device

**Fig. 3:**
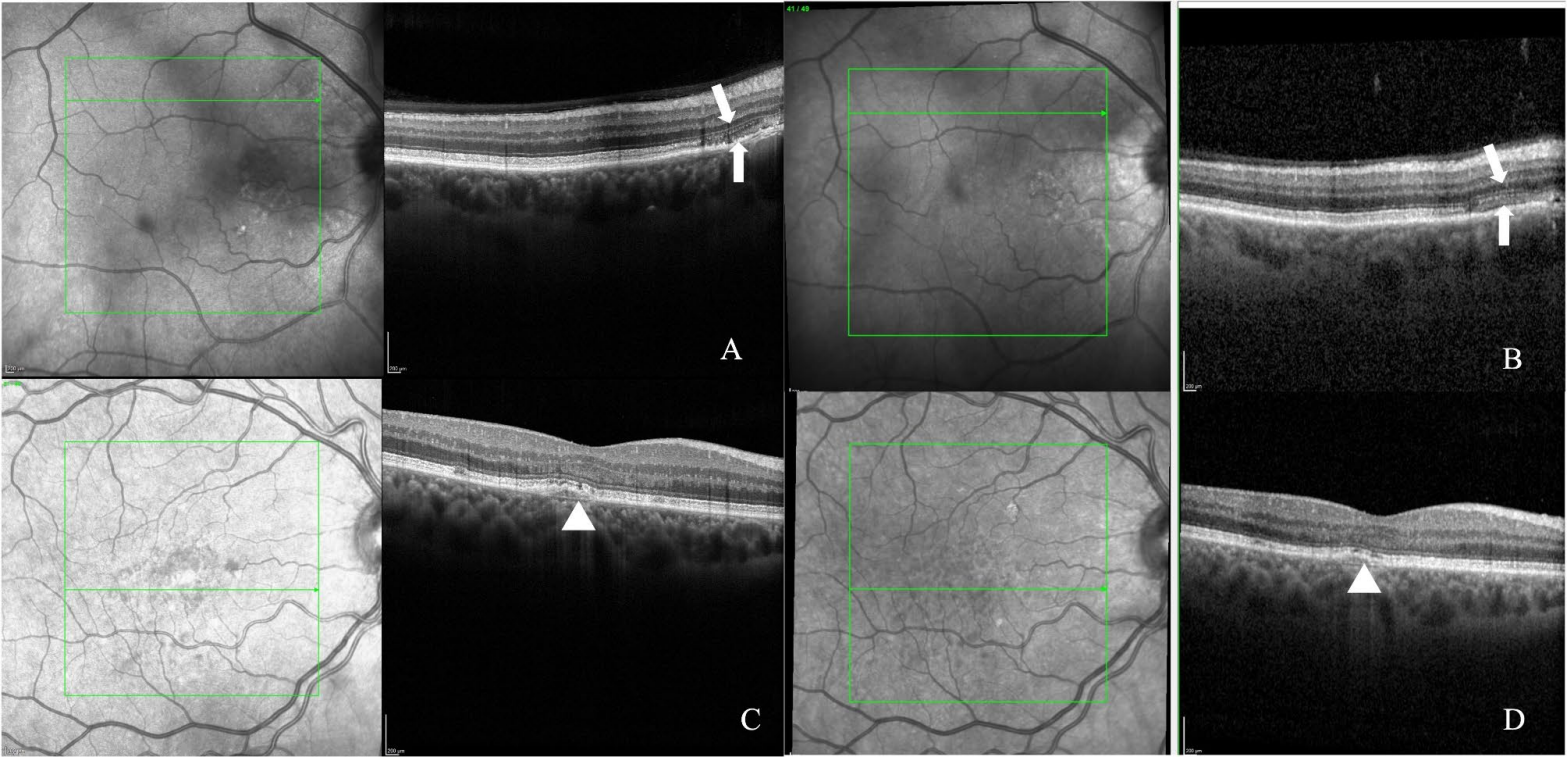
Comparison of ellipsoid zone (EZ) discernibility in two different patients with chronic central serous chorioretinopathy. In the first case High-Resolution OCT **(A)** provides a more detailed visualization of the disrupted EZ, which can be still displayed in standard OCT **(B)** tough less detailed (white arrows). In the second patient, a shallow pigment epithelium detachment (PED) and EZ disruption (white arrow heads) can be visualized both in the High-Resolution acquisition **(C)** and in the standard OCT image **(D)**

High-Resolution OCT revealed a higher prevalence of disrupted IZ, with 29% of cases exhibiting this abnormality compared to only 6% detected by standard OCT (*p* = 0.003). Similarly, High-Res OCT detected disruptions in the RPE layer in a higher percentage of patients, (17%) as compared with 6% reported with the standard OCT modality (*p* = 0.023) **(**Figs. 1 and 2**)** However, the other investigated parameters showed no significant differences between the two modalities, including intact ELM, intact EZ, absence of foveal bulge, absence of flat irregular PED, absence of dome-shaped PED, absence of fibrin exudation, absence of a high reflective band in ONL, absence of hyperreflective foci in ONL, absence of choroidal hyperreflective dots, BM integrity and absence of CME. **(**Fig. 3**) (**Table 2**)** The evaluation of intergrader agreement yielded an average value of 0.85 (Cohen’s Kappa coefficient) across all examined biomarkers in OCT images.

**Table 2:** Comparison of biomarkers detection between standard Spectralis-OCT and high-resolution OCT in patients with central serous chorioretinopathy

Biomarker	Standard OCT (%)	High-Res OCT (%)	*p* value
Disrupted ELM	14 (45.2)	14 (45.2)	1.0
Disrupted Ellipsoid	23 (74.2)	22 (71.0)	0.766
Disrupted IZ	25 (80.6)	29 (93.5)	**0.003**
Absence of foveal bulge	12 (38.7)	10 (32.3)	0.596
RPE protuberance in SRF cavity	11 (35.4)	12 (38.7)	**0.671**
Disrupted RPE	6 (19.4)	14 (45.2)	**0.023**
Disrupted BM	0 (0.0)	0 (0.0)	1.0
Flat irregular PED	16 (51.6)	17	0.799
Dome-shaped PED	10 (32.3)	10	1.0
Fibrin exudation	16 (51.6)	16 (51.6)	1.0
High reflective band in ONL	3 (9.7)	7 (22.6)	0.167
HF in ONL	27(87.1)	28 (90.3)	0.102
Choroidal HF	19 (61.3)	23 (74.2)	0.277
CME	3 (9.7)	4 (12.9)	0.688

BM = Bruch membrane; CME = Central macualr edema; ELM = External limiting membrane; HF = Hyperreflective foci; IZ = Interdigitation zone; ONL = Outer nuclear layer; PED = Pigment epithelium Detachment; RPE = Retinal pigment epithelium; SRF = Subretinal fluid

## Discussion

High-Resolution OCT represents a significant advancement in retinal imaging technology, offering superior axial resolution compared to standard OCT platforms. In OCT, axial resolution is characterized by two pivotal factors: the central wavelength and the bandwidth of the light source. Specifically, resolution increases proportionally to the square of the central wavelength and inversely with the bandwidth of the source. The High-Res OCT device optimally adjusts these parameters by reducing the central wavelength from 880 nm to 853 nm while simultaneously widening the bandwidth from 50 nm to 137 nm. This meticulous fine-tuning of resolution parameters augurs well for enhanced visualization of retinal structures and the discernment of subtle biomarkers linked with various retinal pathologies; however, while the High-Resolution OCT provides superior image quality and finer detail, it requires a longer acquisition time compared to the standard OCT, which may impact patient comfort and feasibility in clinical settings [8, 11] .

In our study we demonstrated that High-Resolution was superior to standard OCT in detecting abnormalities occurring in CSC at the level out the outer retinal layers, including the disruption of IZ, RPE and RPR protuberance in SRF cavity. While these findings underscore the potential of High-Resolution OCT as a valuable diagnostic tool for CSC, further scrutiny is necessary to fully understand its clinical relevance and impact on patient management.

Currently it is imperative to acknowledge the limitations in our understanding of CSC pathology, primarily due to the absence of histological studies in human maculas and the inadequacy of animal models that accurately replicate the macular anatomy [3]. Histological studies are paramount for describing the precise cellular and subcellular changes underlying CSC. Nevertheless, High-Resolution OCT serves as a crucial tool in bridging this gap by offering enhanced visualization at the cellular and subcellular levels of retinal structures in vivo.

In this regard, our group showed in a prospective study on healthy subjects an enhanced visualization at the cellular and subcellular levels of retinal structures using High-Resolution OCT. This included the clear delineation of cell nuclei in ganglion cells and distinct features of photoreceptor and RPE cells, highlighting its superiority over standard OCT in the study subjects [3]; however, to date, no studies have investigated its application in patients with CSC.

Previously, several studies have investigated by standard OCT the alterations occurring in the outer retinal layers along with CSC clinical course, including structural changes at the level of EZ and IZ and the thinning of the ONL, following the reabsorption of SRF reabsorption [12–14]. Furthermore, the ONL thickness has been reported as aa strong predictor of visual prognosis in patients with CSC [15–18]. The current understanding of CSC pathogenesis highlights the pivotal contribution of the underlying choroid in nourishing the outer retinal layers. In fact, in CSC this dynamic interplay is dysregulated due to choroidal ischemia, precipitating photoreceptor apoptosis and consequent disruption, and thinning of the photoreceptor structure [19, 20]. Animal models have shown that SRF-induce retinal detachment is resulting in photoreceptor loss within just 1 to 3 days after the event, aligning with present evidence in CSC and underscoring that the disruption of the outer retinal layers is occurring in the early stages of the disease [21, 22].

In our study, High-Resolution OCT detected RPE changes in 45% of the patients, compared to 19% with standard OCT. This enhanced capability in identifying microstructural alterations at the level of the RPE holds significant clinical implications. In this regard, focal RPE breaks have been shown in patients with CSC, especially occurring at the level of the point of leak visible at FA imaging [23]; these focal RPE microrips has been postulated to be responsible for fluid passage in the subretinal space from the underlying hyperemic choroid [24]. In fact, it may suggest the potential adoption of this device within the early diagnostic framework of the disease, facilitating more precise identification of leakage points and guiding ICGA-FA guided-PDT therapy. We deem that future research should aim to integrate high-resolution OCT findings with ICGA and FA results to further assess its utility in managing patients with CSC.

Secondly, our study demonstrated that while High-Resolution OCT did not outperform the standard OCT in detecting microstructural changes at the ELM and EZ levels in CSC patients, it notably was superior in capturing subtle alterations at the IZ level. This is significant because the IZ serves as a crucial prognostic biomarker for visual function in both healthy aging subjects and those with macular diseases [25]; however, the discernibility of this layer is not consistently achievable with standard OCT. In this context, Berlin et al. observed in a cohort of healthy individuals that the IZ was more readily discernible in younger eyes compared to older ones [26]. Despite this age-related difference, its presence correlated positively with faster dark adaptation, suggesting its potential as a biomarker indicative of visual function throughout the aging process [27]. Thus, we postulate that the higher capability of High-Resolution OCT as compared with standard OCT to detect disruptions in the IZ may enhance the possibility for clinicians to predict long-term visual outcomes in patients with CSC. Nonetheless, further validation through larger-scale studies is essential to consolidate these findings.

This study has some limitations, including the small sample size, its non-randomized nature, and the absence of a long follow-up period. Moreover, the choroid plays an important role in the pathophysiology of CSC and can be visualized using OCT, although details of such visualization apart from the choroidal thickness remain incompletely understood. Choroidal anatomy and features were not explored in this study specifically but may be a relevant topic for future studies.

In conclusion, our study highlights the superior performance of High-Resolution OCT as compared with standard OCT in detecting key imaging biomarkers associated with CSC, particularly disruptions in the IZ and RPE. This is mainly due to and increased axial resolution allowing for more precise of hyperreflective structures (such as the IZ and RPE), as well as subtle hyporreflective changes with the SRF cavity. This dual capability of better resolving both types of reflectivity contributed significantly to High-Resolution superior diagnostic performance.

While promising, further research is needed to validate these findings in larger cohorts and assess the clinical implications of High-Resolution OCT in predicting long-term visual outcomes for patients with CSC.

## Data Availability

All data are available and kept in Inselspital protected database.

## References

[1] Feenstra HMA, van Dijk EHC, Cheung CMG et al. Central serous chorioretinopathy: an evidence-based treatment guideline. Prog Retin Eye Res 2024:101236. 10.1016/j.preteyeres.2024.101236 38301969

[2] Fung AT, Yang Y, Kam AW. Central serous chorioretinopathy: a review. Clin Exp Ophthalmol. 2023;51(3):243–70. 36597282 10.1111/ceo.14201

[3] Daruich A, Matet A, Dirani A, et al. Central serous chorioretinopathy: recent findings and new physiopathology hypothesis. Prog Retin Eye Res. 2015;48:82–118. 26026923 10.1016/j.preteyeres.2015.05.003

[4] Nkrumah G, Paez-Escamilla M, Singh SR, et al. Biomarkers for central serous chorioretinopathy. Ther Adv Ophthalmol. 2020;12:2515841420950846. 32923941 10.1177/2515841420950846 PMC7448152

[5] Desideri LF, Scandella D, Berger L, Sznitman R, Zinkernagel M, Anguita R. Prediction of chronic central serous chorioretinopathy through combined manual annotation and AI-assisted volume measurement of flat irregular pigment epithelium. Ophthalmologica 2024. 10.1159/000538543 38555632

[6] Yoneyama S, Fukui A, Sakurada Y, et al. Distinct characteristics of simple Versus Complex Central Serous Chorioretinopathy. Retina. 2023;43(3):389–95. 36729824 10.1097/IAE.0000000000003692

[7] Rothenbuehler SP, Malmqvist L, Belmouhand M et al. Comparison of spectral-domain OCT versus swept-source OCT for the detection of Deep Optic Disc Drusen. Diagnostics (Basel). 2022;12(10). 10.3390/diagnostics12102515 PMC9600200 36292204

[8] Reche J, Stocker AB, Henchoz V, et al. High-resolution optical coherence tomography in healthy individuals provides Resolution at the Cellular and subcellular levels. Transl Vis Sci Technol. 2023;12(7):12. 37428129 10.1167/tvst.12.7.12 PMC10341292

[9] Ferro Desideri L, Barra F, Ferrero S. Methodological concerns on retinal and choroidal thickness variations measured by optical coherence tomography in patients with epilepsy. Epilepsy Behav. 2019;94:312. 30982684 10.1016/j.yebeh.2019.02.027

[10] Singh SR, Iovino C, Zur D, et al. Central serous chorioretinopathy imaging biomarkers. Br J Ophthalmol. 2022;106(4):553–8. 33288526 10.1136/bjophthalmol-2020-317422

[11] Habra O. High resolution optical coherence tomography in patients with age related macular degeneration. IOVS ARVO Abstract.; 2022. https://iovs.arvojournals.org/article.aspx?articleid=2782799.

[12] Matsumoto H, Sato T, Kishi S. Outer nuclear layer thickness at the fovea determines visual outcomes in resolved central serous chorioretinopathy. Am J Ophthalmol. 2009;148(1):105–10. e101. 19327740 10.1016/j.ajo.2009.01.018

[13] Ratanasukon M, Thongthong K, Bhurayanontachai P, Jirarattanasopa P. Photoreceptor disruption in central serous chorioretinopathy treated by half-dose photodynamic therapy. Clin Ophthalmol. 2013;7:87–92. 23345962 10.2147/OPTH.S39584 PMC3548438

[14] Asano KS, Asaoka R, Asano S, Azuma K, Inoue T, Obata R. Elongated photoreceptor outer segment length and prognosis of Chronic Central Serous Chorioretinopathy. Retina. 2020;40(4):750–7. 30640283 10.1097/IAE.0000000000002445

[15] Fujita K, Imamura Y, Shinoda K, et al. One-year outcomes with half-dose verteporfin photodynamic therapy for chronic central serous chorioretinopathy. Ophthalmology. 2015;122(3):555–61. 25444637 10.1016/j.ophtha.2014.09.034

[16] Nicholson BP, Ali Idris AM, Bakri SJ. Central Serous Chorioretinopathy: clinical characteristics Associated with visual outcomes. Semin Ophthalmol. 2018;33(6):804–7. 30067427 10.1080/08820538.2018.1503690

[17] Torres-Costa S, Penas S, Cerqueira AR, et al. Long term outer retinal changes in central serous chorioretinopathy submitted to half-dose photodynamic therapy. Photodiagnosis Photodyn Ther. 2021;34:102235. 33631379 10.1016/j.pdpdt.2021.102235

[18] Piccolino FC, de la Longrais RR, Ravera G, et al. The foveal photoreceptor layer and visual acuity loss in central serous chorioretinopathy. Am J Ophthalmol. 2005;139(1):87–99. 15652832 10.1016/j.ajo.2004.08.037

[19] Paques M, Tadayoni R, Sercombe R, et al. Structural and hemodynamic analysis of the mouse retinal microcirculation. Invest Ophthalmol Vis Sci. 2003;44(11):4960–7. 14578423 10.1167/iovs.02-0738

[20] Wong RL, Singh SR, Rasheed MA, et al. En-face choroidal vascularity in central serous chorioretinopathy. Eur J Ophthalmol. 2021;31(2):536–42. 32103680 10.1177/1120672120908719

[21] Anderson DH, Guerin CJ, Erickson PA, Stern WH, Fisher SK. Morphological recovery in the reattached retina. Invest Ophthalmol Vis Sci. 1986;27(2):168–83. 3943943

[22] Rex TS, Fariss RN, Lewis GP, Linberg KA, Sokal I, Fisher SK. A survey of molecular expression by photoreceptors after experimental retinal detachment. Invest Ophthalmol Vis Sci. 2002;43(4):1234–47. 11923271

[23] Gupta V, Gupta P, Dogra MR, Gupta A. Spontaneous closure of retinal pigment epithelium microrip in the natural course of central serous chorioretinopathy. Eye (Lond). 2010;24(4):595–9. 19648895 10.1038/eye.2009.193

[24] Hirami Y, Tsujikawa A, Sasahara M, et al. Alterations of retinal pigment epithelium in central serous chorioretinopathy. Clin Exp Ophthalmol. 2007;35(3):225–30. 17430508 10.1111/j.1442-9071.2006.01447.x

[25] Subhi Y, Forshaw T, Sorensen TL. Macular thickness and volume in the elderly: a systematic review. Ageing Res Rev. 2016;29:42–9. 27262495 10.1016/j.arr.2016.05.013

[26] Kalra G, Cetin H, Whitney J et al. Automated identification and segmentation of Ellipsoid Zone At-Risk using deep learning on SD-OCT for Predicting Progression in Dry AMD. Diagnostics (Basel). 2023;13(6). 10.3390/diagnostics13061178 PMC10047385 36980486

[27] Berlin A, Matney E, Jones SG, et al. Discernibility of the Interdigitation Zone (IZ), a potential optical coherence tomography (OCT) biomarker for visual dysfunction in aging. Curr Eye Res. 2023;48(11):1050–6. 37539829 10.1080/02713683.2023.2240547 PMC10592305

